# The Italian version of the Majeed pelvic score: translation, cross-cultural adaptation and validation

**DOI:** 10.1007/s12306-024-00858-6

**Published:** 2024-11-11

**Authors:** G. Vittone, S. Cattaneo, C. Galante, M. Domenicucci, M. F. Saccomanno, G. Milano, A. Casiraghi

**Affiliations:** 1 Department of Medical and Surgical Specialties, Radiological Sciences, and Public Health, University of Brescia, Viale Europa 11, 25123 Brescia, BS Italy; 2 Department of Bone and Joint Surgery, ASST Spedali Civili, Piazzale Spedali Civili 1, 25123 Brescia, BS Italy

**Keywords:** Majeed pelvic score, Pelvic ring fracture, Questionnaire, Patient-reported outcome measurements

## Abstract

**Purpose:**

The assessment of functional outcomes after pelvic ring fracture remains a controversial topic. The Majeed pelvic score (MPS) is the most commonly used pelvic-specific questionnaire in the literature. The aim of this study is translation, cross-cultural adaptation and validation of the Italian version of MPS.

**Methods:**

The study was articulated in two phases. Phase 1 consisted in translation and cross-cultural adaptation of MPS, from English into Italian. The psychometric properties were tested on 52 Italian patients (Phase 2). Construct validity was assessed by correlation with Short-Form 12 (SF-12). 33 patients repeated the questionnaire after 14 days to assess its reproducibility. All data were subsequently analyzed (descriptive statistics, multitrait analysis, reliability and construct validity assessment).

**Results:**

The questionnaire was clear and easily understood (no missing data). A ceiling effect was detected for all items of the scale. Multitrait analysis showed good results for each outcome measure, except for the item “walking distance” that showed poor item discriminant validity. A significant correlation between the MPS and the physical component summary (PCS) of the SF-12 was found, while there was a weak correlation with the mental component summary (MCS). The questionnaire showed high internal consistency (Cronbach’s alpha: 0.91–0.99) and very good test–retest reliability (intraclass correlation coefficients: 0.92–0.96).

**Conclusions:**

The Italian version of the MPS has demonstrated to be reliable and valid in the evaluation of patients with pelvic ring fractures. There is still however a need for an instrument capable of evaluating the mental component in these types of injuries.

**Supplementary Information:**

The online version contains supplementary material available at 10.1007/s12306-024-00858-6.

## Introduction

Pelvic ring fractures represent about 3% of all fractures, with an incidence rate of 19–37 cases every 100.000 people a year [1].

Different classifications, based on fracture patterns and radiological findings, have been developed over the years [2–6]. However, adequate prospective follow-up studies that evaluate functional outcomes after these injuries are still lacking.

It is now widely accepted that patient’s perception represents an important factor when evaluating the impact of an injury and the efficacy of a treatment. The growing focus on patient-centered care has resulted in a shift in terms of outcome assessment and has led to an increase in the use of patient-reported outcome measurements (PROMs). Their function is to aid the clinician in obtaining an objective assessment of the perceived health status of the patient. To be able to perform this task, adequate questionnaires should be standardized, validated and tested to reliably evaluate the parameters they were developed to inquire. Translation of a questionnaire into a different language requires a process of cross-cultural adaptation. A standardized method is needed when performing cross-cultural adaptation of a PROM to ensure maintenance of its validity and reliability into a different cultural setting.

Assessment of pelvic ring function and related quality of life has not yet been standardized and many different generic and pelvis-specific questionnaires have been employed. Among pelvis-specific PROMs, the Majeed pelvic score (MPS) is the most frequently used [7, 8].

The MPS was proposed in an effort to develop an objective method to evaluate functional outcomes after pelvic ring fracture [9]. Although this instrument has never been properly validated in patients with pelvic ring fractures, its frequent use might allow the possibility of comparing outcomes to those of other studies.

The purpose of the present study was the translation and cross-cultural adaptation of the MPS for the Italian-speaking population and its validation for the assessment of functional outcomes after pelvic ring fractures. The hypothesis of the study was that the Italian version of MPS is reliable and valid.

## Methods

### Study design

We designed an observational cross-sectional monocentric study. The study was approved by the Institutional review board and ethical committee of our Institution in accordance with the Helsinki Declaration.

The study was divided into two separate and consequential steps. The first step consisted in the translation and cross-cultural adaptation of the MPS from the original English version into the Italian version. The second step was represented by the validation process.

The process of translation and cross-cultural adaptation was carried out according to the American Academy of Orthopedic Surgeons (AAOS) Outcomes Committee [10]. Validation of the translated questionnaire followed the IQOLA project’s analysis plan [11, 12] and current guidelines [13, 14].

### Study population

Patients were selected from a database comprising all pelvic ring fractures treated in our institution between December 2018 and September 2021.

We analyzed retrospectively patient’s clinical data and radiographic images. Criteria for inclusion in the study were: diagnosis of a pelvic ring fracture (treated either conservatively or surgically), age over 18 y/o, at least 6 months of time elapsed from trauma, the ability to compile questionnaires in complete autonomy and acquisition of informed consent. The presence of neurological disorders, pelvic floor muscle or pelvic organ disorders before the trauma were considered exclusion criteria as well as the inability to compile the questionnaires due to linguistic barriers or cognitive disorders.

### Translation and cross-cultural adaptation

The translation and cross-cultural adaptation were articulated into different phases.

The first phase consisted of independent translation of the original version of the MPS by two bilingual translators (Italian and American English). One translator was an orthopedic specialist, the other one was a professional translator.

During the second phase we merged the two translated versions under the supervision of the research coordinator (an expert orthopedic pelvic surgeon). Discrepancies between the two versions were discussed and a unified translation was obtained (Version 1).

In the third phase a back translation of Version 1 was performed by two different American English native speaker translators, not privy to the original version of the MPS. This was done to ensure that the content of the original questionnaire was not altered by the translation.

During the fourth phase a research committee composed by the scientific director, the research coordinator, the four translators, two epidemiologists, a statistician and an Italian linguist paired the two back translations with the original version of the MPS. A detailed report of the meeting was filled, and the critical points were discussed until a consensus was achieved. After this meeting a pre-final version of the translated MPS was created.

After obtaining consent the pre-final version was administered to 30 patients considered eligible for the study. The sample size was chosen in accordance to current guidelines [10, 15]. After completing the questionnaire each patient was asked about any difficulty found during the compilation and the findings were then discussed by an expert committee and addressed. A final version of the translated MPS was then drafted and approved by the authors.

### Validation of the final version

In this phase 52 patients were recruited; the sample size was calculated according to current guidelines [14, 16]. During the first visit, consent was obtained from all patients and general demographic data was recorded by a physician (age, sex, ethnicity, age at the time of the trauma, and occupation). The patients were then administered the final version of the translated MPS and the Short Form-12 (SF-12) questionnaire.

The MPS contains five domains composed by a total of seven separate items. The five domains are pain (0–30 points), work (0–20 points), sitting (0–10 points), sexual intercourse (0–4 points), standing (0–36 points). The “standing” domain includes 3 different items (each accounting for a maximum of 12 points): walking aids, gait unaided and walking distance. The maximum score that can be obtained is 100 points for patients working before the injury, while in patients that were not working before the injury the maximum score is 80 points. The original author did not provide a clear guideline for scoring the most severe level of dysfunction in each item, and no standardized method is currently available in literature [17]. The first category of each item was originally presented as a range, but no information as to how to assign a number within that range is currently available. In an effort to try and standardize the results, in our study the scoring of the first category was fixed statically at the highest value for each item.

The SF-12 questionnaire is a multi-item scale routinely used to assess the quality of life and represents a shortened version of the SF-36. It is comprised of 12 items that allow the creation of two synthetic indexes to evaluate the mental and physical health of the subject, respectively the mental component summary (MCS-12) and the physical component summary (PCS-12). A lower score corresponds to a worse health status of the patient. After completion of the questionnaires, 33 patients were randomly selected among the participants and they were asked to compile the MPS a second time after 14 days (retest); the number was sufficient for the scale’s validation and determined according to guidelines and recent literature [10, 12, 15].

### Outcome measures and statistical analysis

The validation of the Italian version of the MPS was performed in accordance with the analysis plan of the IQOLA project [11, 12] and current guidelines [13, 14]. After completing the translation process, the validity of the translated final version was assessed.

All the data were analyzed by SPSS 25 software (IBM Statistics, Harmonk, NY, USA).

Percentage and distribution of missing data, distribution of the answers, mean and standard deviation were reported for every question. Mean and standard deviation were reported for each domain, lower and upper bound answer percentages were calculated for every scale, to identify ceiling and floor effect. Ceiling and floor effect were considered present when reaching 15% of the sample size for the highest and lowest possible score, respectively [14, 18].

Content validity was evaluated by multitrait analysis to assess the correlation between every single question and its hypothetic domain and the other domains. Item internal consistency was evaluated using Pearson’s correlation coefficient. Equality of item scale correlation and item discriminant validity were assessed.

The correlation between the final translated version of the MPS and a commonly used generic indicator of quality of life such as the SF-12 questionnaire was verified to test construct validity. The correlation was considered very weak if the correlation coefficient was 0.0–0.19, weak 0.20–0.39, moderate 0.40–0.69, strong 0.70–0.89, and very strong > 0.9 [19].

Reliability of the questionnaire was assessed by internal consistency analysis and test–retest. Cronbach’s alpha coefficient measured internal consistency for every domain. Internal consistency higher than 0.70 indicates good reproducibility [20]. Intraclass correlation coefficient was adopted to assess test–retest reliability.

## Results

In phase 1, neither linguistic nor lexical problems were detected. Hence no translation problems were detected. No major difficulties in comprehension were revealed during testing of the pre-final version. Data analyzed in phase 2 were collected from 52 patients. There were 31 men (59.6%) and 21 women (40.4%). The average age was 48.2 ± 17.2 years (range 20–80 years). The average follow-up was 20.7 ± 9.8 months (range 6–39 months).

### Descriptive statistics

Distribution of responses for each item are reported in Table 1. There were no missing data. Patients used all the available response options except for three options in the items “walking aids” and “gait unaided”.

**Table 1:** Item descriptive statistics

Scale	Missing (%)	Mean	SD	*Response values frequency*					
				***0–5***	***10***	***15***	***20***	***25***	***30***
Pain	0	20.4	8	4	4	14	6	10	14
				***0–4***	***8***	***12***	***16***	***20***	
Work	0	11.5	6.8	19	5	5	9	14	
				***0–4***	***6***	***8***	***10***		
Sitting	0	8.1	2	4	13	11	24		
				***0–1***	***2***	***3***	***4***		
Sexual intercourse	0	3.3	1.1	6	5	9	32		
**Standing**				***0–2***	***4***	***6***	***8***	***10***	***12***
Walking aids	0	11.6	1.3	0	0	2	1	3	46
Gait unaided	0	10.5	2.6	3	0	2	4	11	32
Walking distance	0	9.8	3.3	1	8	3	3	4	33

A ceiling effect was observed for all items, while floor effect was observed only in the “Work” domain. Overall, the highest score for the MPS was achieved in 6 out of 52 patients (11.5%) showing no significant ceiling effect (Table 2).

**Table 2:** Descriptive statistics for scales (raw scores)

Scale	Observed values						
	Mean	SD	Lowest	Highest	Range	% at floor	% at ceiling
Pain (0–30 pts)	20.4	8	5	30	25	7.7	26.9
Work (0–20 pts)	11.5	6.8	4	20	16	36.5	26.9
Sitting (0–10 pts)	8.1	2	4	10	6	7.7	46.2
Sexual intercourse (0–4 pts)	3.3	1.1	1	4	3	11.5	61.5
Standing (0–36 pts)	31.9	5.9	12	36	24	1.9	51.9

### Content validity

In the MPS only the domain “Standing” is composed by multiple items. Therefore, multitrait analysis was performed only for this scale. Table 3 shows moderate item-scale correlation for all the items. Correlations were 0.40 or more after overlap correction.

**Table 3:** Item internal consistency for standing scale. Item-scale correlation corrected for overlap (relevant item removed from its scale for correlation)

Items	Scales				
	Pain	Work	Sitting	Sexual intercourse	Standing
Walking aids	0.198	0.291*	0.163	−0.023	0.449*
Gait unaided	0.407*	0.416*	0.325*	0.106	0.662*
Walking distance	0.518*	0.545*	0.440*	0.344*	0.498*

*Statistically significant correlation

Each item contributed in the same measure to the overall score in every domain granting equality of item-scale correlation.

The correlation between each item and its own domain was generally higher in comparison with other domains although it was not significant in all cases. The only exception was “Walking distance” that showed higher correlation with “Pain”, “Work” and “Sitting” although not significantly (Table 4). Overall, item-scale correlation was higher for hypothesized scale than for competing scales in 75% of the matched comparisons (Table 5).

**Table 4:** Item discriminant validity tests for standing scale

Items	Scales				
	Pain	Work	Sitting	Sexual intercourse	Standing
Walking aids	1	1	2	2	**
Gait unaided	1	1	2	2	**
Walking distance	−1	−1	−1	1	**

Cutoff point for significance is 2 standard errors

Levels of scaling success:

2: Item-scale correlation is significantly higher for hypothesized scale than for competing scale

1: Item-scale correlation is higher for hypothesized scale than competing scale, but not significantly

−1: Item-scale correlation is lower for hypothesized scale than competing scale, but not signficantly

−2: Item-scale correlation is significantly lower for hypothesized scale than for competing scale

**Discriminant validity test not conducted

**Table 5:** Frequency and percentage of Item-scale correlations at each level of scaling success for standing scale

Scale	−2		−1		1		2		1 + 2	
	*n*	*%*	*n*	*%*	*n*	*%*	*n*	*%*	*n*	*%*
Standing	0	0	3	25	5	41.7	4	33.3	9	75

Levels of scaling success:

2: Item-scale correlation is significantly higher for hypothesized scale than for competing scale

1: Item-scale correlation is higher for hypothesized scale than competing scale, but not significantly

−1: Item-scale correlation is lower for hypothesized scale than competing scale, but not signficantly

−2: Item-scale correlation is significantly lower for hypothesized scale than for competing scale

### Construct validity

The MPS was correlated with the PCS and MCS scales of the SF-12 questionnaire for construct validity assessment. The calculations were made on the whole sample (Table 6). Each domain of MPS showed a significant correlation with the PCS. Conversely, only “sexual intercourse” domain of MPS showed a significant correlation with the MCS.

**Table 6:** Correlation between Majeed score and SF-12 (Physical and mental component summaries)

Scales	SF-12 (PCS)		SF-12 (MCS)	
	*r*	*p*	*r*	*p*
Pain	0.830	< 0.0001*	0.048	0.736
Work	0.570	< 0.0001*	0.187	0.183
Sitting	0.601	< 0.0001*	0.002	0.990
Sexual intercourse	0.520	< 0.0001*	0.330	0.017*
Standing	0.590	< 0.0001*	0.041	0.771
Overall	0.853	< 0.0001*	0.121	0.391

*Statistically significant correlation

### Reliability

Cronbach’s alpha in the final version of the MPS ranged from 0.924 (standing) to 0.953 (work) (Table 7). Reliability at test re-test was excellent, with ICCs ranging from 0.921 for “sexual intercourse” to 0.954 for “work” (Table 8).

**Table 7:** Reliability coefficients and inter-scale correlations at test–retest evaluation

Scales	Pain	Work	Sitting	Sexual intercourse	Standing	Overall
Pain	0.927	0.474	0.530	0.273	0.805	0.722
Work	0.646	0.953	0.443	0.179	0.663	0.583
Sitting	0.429	0.362	0.926	0.521	0.499	0.249
Sexual intercourse	0.254	0.137	0.553	0.926	0.165	0.104
Standing	0.754	0.633	0.489	0.209	0.924	0.610
Overall	0.756	0.537	0.283	0.119	0.706	0.969

Scale internal consistency reliability (Cronbach’s alpha coefficient) is presented in the diagonal (cells with underlined text)

**Table 8:** Intraclass correlation coefficients at test–retest evaluation

Scales	*ICC*	95% CIs	
		Lower limit	Upper limit
Pain	0.930	0.855	0.966
Work	0.954	0.906	0.978
Sitting	0.927	0.852	0.964
Sexual intercourse	0.921	0.837	0.962
Standing	0.922	0.840	0.962
Overall	0.969	0.937	0.985

ICC, intraclass correlation coefficient

## Discussion

Reporting results and outcomes in orthopedics and traumatology has historically been focused on radiographic measures and objective assessment of function. However, evaluation of subjective health status has gained increasingly more importance, both in clinical practice and in scientific research. This shift in outcome assessment, with increasing emphasis on patient-reported outcome measures (PROMs), has led to the need of instruments that are validated, reliable and reproducible. PROM-based assessment after pelvic ring injuries is still not standardized and many different types of generic outcome measure instruments as well as pelvis-specific measures are currently used. Lefaivre et al. [7] and later on Banierink et al. [8] performed systematic reviews on this subject. In both reviews the Majeed Pelvic Score (MPS) was the most used pelvis-specific instrument. Indeed, limited testing of its psychometric properties has been done in the past, and only a few number of studies evaluating construct validity, content validity and reliability of this score [21–26]. However, its widespread use make it the best way to compare outcomes of different studies and data sets [17].

In our study we performed a translation and cross-cultural adaptation into the Italian language of the Majeed pelvic score and we determined its psychometric properties in the evaluation of functional outcomes after pelvic ring fractures.

In phase 1 during the translation and cross-cultural adaptation process, much attention was given to the straightforwardness of the text and no major contention point was highlighted. We focused on using simple terms that are in common use, making it easily understandable. We think brevity, coupled with simple wording, allowed for a high completion rate, with no missing data to report. Among the studies evaluating the MPS’ psychometric properties there are two instances of translation, cross-cultural adaptation and measurement of the psychometric properties of the translated MPS: one in the Lithuanian language by Petryla et al. [23] and one in the Italian language by Busso et al. [22] In both cases the brevity of the MPS and its intuitive formulation allowed translation without any reported issue. At the beginning of our work no translation and validation of the MPS into the Italian language had been previously published. Busso et al. [22], in their work, translated the MPS into the Italian language following the same step by step process as our group did. However, there are some key differences between our work and that by Busso et al. [22]. First, in our work the MPS was tested on 52 patients, while Busso et al. [22] included only 21 patients in their analysis. Indeed, a larger sample size might help giving a more accurate depiction of the questionnaire’s properties in the general population. A second difference is the type of pathology analyzed. We chose to extend testing of the questionnaire on all pelvic ring fracture patients, regardless of fracture and treatment type, while Busso et al. [22] tested the psychometric properties of the translated Italian version of the MPS and Iowa pelvic score (IPS) on patients who underwent surgical sacroiliac joint arthrodesis after fracture. Third, construct validity testing differed in the two studies. We analyzed the correlation with the SF-12 questionnaire, a shorter version of the SF-36. In Busso et al.’s [22] work construct validity was tested by correlation with the Italian Owestry disability index (ODI) [27] a validated pathology-specific score for lower back pain that showed acceptable correlation with the MPS in analyzing sacroiliac joint pain [28]. A strong correlation between the Physical Component Summary (PCS) of the SF-36 and the MPS has been well documented in literature [21, 23–26], indicating that the questionnaire has good properties of assessing the physical outcomes of the injury. In our study a strong correlation was confirmed with the PCS of the SF-12. Conversely, MPS showed no significant correlation with the MCS, except for the “sexual intercourse” item. The MPS is a score designed to explore function, not psychological and emotional state, thus it is not surprising that no correlation has been found with MCS. Similarly, LeFaivre et al. [21] reported a weak correlation between the mental component of the SF-36 and both MPS and all other pelvis-specific instruments evaluated. They concluded that these questionnaires are failing to capture elements that affect psychological distress and limitations in usual social or work activities due to emotional problems.

A fourth difference between our work and Busso et al.’s [22] is represented by scoring of the most severe level of dysfunction in each item. In our study scoring was fixed statically at the highest value for each item, while in Busso et al.’s work it was fixed at the lowest. In a recent systematic review, Kleweno et al. [17] analyzed the inaccuracies in the use of the MPS. In their work they found no article discussing how the range of possible scores available for the most severe function was applied in the calculation of the published overall outcome scores. In one of the studies analyzed [29], however, the authors fixed the scoring at the highest value for each category in a similar way to ours. As the original author did not provide a clear guideline on how to assign a number within the available range, and no standardized method is currently available [17], we cannot state that either method is better than the other, but we felt that using the lowest possible value might represent too much of a drop-off on the overall score.

We found a significant ceiling effect (over 15%) in all domains of the translated MPS, in accordance with the existing literature [21–23, 28]. This ceiling effect has been shown by Brouwers et al. [30] to be present already 3 months after injury. Busso et al. [22] reported a ceiling effect of the overall questionnaire at a minimum of 5 years of follow-up. Petryla et al. [23] observed that ceiling effect wasn’t present 2 months after injury, while it appeared in a second evaluation at 12 months. This may be explained by muscle repair and bone healing and their influence on pain, physical activity and walking performance. The ceiling effect indicates a scarce ability of the MPS to differentiate between outcome changes at different time intervals, at midterm and long-term follow-up.

The “work” domain showed a floor effect that was not reported in previous articles. This might be due to an erroneous comprehension of the question by the patients or to an intrinsic difficulty in the interpretation given the substantial variation in job duties. A relatively sedentary desk job cannot be compared with a manual labor and thus the return to job function is difficult to compare [17].

Overall, the items showed a higher correlation with their domain compared to the others, indicating good item discriminant validity. The only exception was for “walking distance”. This item showed a higher correlation, although not statistically significant, with “pain”, “work” and “sitting” scales. Inclusion of an item that failed the test of item discriminant validity due to low internal consistency might complicate the interpretation of the scale and render the questionnaire less efficient [12]. Failing of this test might be due to a misinterpretation occurred during the translation process and as such a retranslation of this item or exclusion from the scale might be considered in future studies. Internal consistency analysis showed good reproducibility with a Cronbach’s alpha of 0.939 and test–retest reliability was excellent, with ICCexceeding 0.90 for all the domains, thus confirming the findings of Busso et al. [22].

This study has some limitations. The examined sample was large enough to reach the validation of the MPS Italian version; however, the study group might not be large enough to represent overall the population of interest. Moreover, we wanted to apply the questionnaire to all pelvic fracture patients, as proposed by Majeed [9] in the original formulation of the questionnaire, but no stratification of patients according to fracture classification or associated injuries has been made.

## Conclusions

The Italian version of MPS has proved to be reliable and valid in assessing physical outcomes of pelvic ring fracture patients, although it still lacks capabilities in capturing the psychological component that is commonly linked with this type of injuries. Moreover, the high ceiling effect of the MPS may limit its ability in stratifying patients in the long-term follow-up. There still is a need of a comprehensive instrument able to evaluate both the physical and mental component that are intertwined in patients with pelvic ring fractures.

## Supplementary Information

Below is the link to the electronic supplementary material.

Supplementary file1 (DOCX 20 KB)
