# Supplementary material for: The Italian version of the Majeed pelvic score: translation, cross-cultural adaptation and validation
Source: Musculoskelet Surg. 2024 Nov 11;109(2):215–22. doi: 10.1007/s12306-024-00858-6 (PMC12122645; doi:10.1007/s12306-024-00858-6)
Supplement: Supplementary file 1 — Supplementary file1 (DOCX 20 KB) [file 12306_2024_858_MOESM1_ESM.docx]

**Majeed score**

1. **Dolore - 30 punti**

| Intenso e continuo a riposo | 0-5 |
| --- | --- |
| Intenso durante l’attività | 10 |
| Sopportabile ma limita l’attività | 15 |
| Con attività moderata, abolito dal riposo | 20 |
| Moderato, intermittente, normali attività | 25 |
| Lieve, occasionale o assente | 30 |

1. **Lavoro - 20 punti**

| Nessun lavoro regolare | 0-4 |
| --- | --- |
| Lavoro leggero | 8 |
| Cambio di lavoro | 12 |
| Stesso lavoro, ridotto rendimento | 16 |
| Stesso lavoro, stesso rendimento | 20 |

1. **Stare seduti - 10 punti**

| Doloroso | 0-4 |
| --- | --- |
| Doloroso se prolungato o impacciato | 6 |
| Fastidioso | 8 |
| Libera | 10 |

1. **Rapporto sessuale - 4 punti**

| Doloroso | 0-1 |
| --- | --- |
| Doloroso se prolungato o impacciato | 2 |
| Fastidioso | 3 |
| Libero | 4 |

1. **Stare in piedi - 36 punti.**

**(A) Ausili per la deambulazione (12)**

| Costretto a letto o quasi | 0-2 |
| --- | --- |
| Sedia a rotelle | 4 |
| Due stampelle | 6 |
| Due bastoni | 8 |
| Un bastone | 10 |
| Senza bastone | 12 |

**(B) Deambulazione senza ausili (12)**

| Non può camminare o quasi | 0-2 |
| --- | --- |
| Trascinando piccoli passi | 4 |
| Zoppia grave | 6 |
| Zoppia moderata | 8 |
| Zoppia lieve | 10 |
| Normale | 12 |

**(C) Distanza camminata (12)**

| Costretto a letto o pochi metri | 0-2 |
| --- | --- |
| Tempo e distanza molto limitati | 4 |
| Limitata con bastoni, difficile senza, stazione eretta prolungata possibile | 6 |
| Un’ora con un bastone, limitata senza | 8 |
| Un’ora senza bastoni, con lieve dolore o zoppia | 10 |
| Normale per età e condizioni generali | 12 |

| **Lavorava prima dell’infortunio** | **Non lavorava prima dell’infortunio** | **Categoria** |
| --- | --- | --- |
| >85 | >70 | Eccellente |
| Da 70 a 84 | Da 55 a 69 | Buono |
| Da 55 a 69 | Da 45 a 54 | Sufficiente |
| <55 | <45 | Scarso |
